# Pharmacologic Means of Extending Lifespan

**DOI:** 10.4172/2161-0681.S4-002

**Published:** 2012-05-17

**Authors:** Dudley W. Lamming, David M. Sabatini, Joseph A. Baur

**Affiliations:** 1 Department of Biology, MIT, Cambridge, MA 02139, Howard Hughes Medical Institute, MIT, Cambridge, MA 02139; Whitehead Institute for Biomedical Research, Cambridge MA 02142, Broad Institute of Harvard and MIT, Seven Cambridge Center, Cambridge, MA 02142, The David H. Koch Institute for Integrative Cancer Research at MIT, Cambridge, MA 02139, USA; 2 Department of Physiology, Institute for Diabetes, Obesity, and Metabolism, Perelman School of Medicine, University of Pennsylvania, Philadelphia PA 19104, USA

## Introduction

Humanity has long dreamed of the possibility of extending lifespan, from the search for the mythical philosopher’s stone to Ponce de Leon’s quest for the Fountain of Youth. While no such fantastical solution exists, recent advances in aging research have brought us closer to finding treatments that may aid in slowing the aging process and attenuating age-related diseases. In this review, we will discuss small molecules that recent evidence suggests may be capable of improving health and extending lifespan in mammals, focusing particularly on resveratrol, rapamycin, and metformin.

## Resveratrol

One of the most frequently mentioned potential anti-aging compounds is resveratrol, a polyphenol found in a variety of plant sources, including berries, peanuts, and red wine [1]. In 2003, the laboratories of Howitz KT et al. [2] showed using an *in vitro* assay that resveratrol and several structurally-related compounds activate SIRT1, an NAD-dependent protein deacetylase that is homologous to the product of the yeast longevity gene Silent Information Regulator 2 (*SIR2*) [2]. Furthermore, resveratrol was subsequently shown to extend the lifespan of yeast, and to confer stress-resistance to cultured mammalian cells. Later studies expanded these findings to show resveratrol-dependent lifespan extension in *Caenorhabditis elegans, Drosophila melanogaster* [3], and a short-lived species of vertebrate fish [4].

The initial trial of resveratrol’s effects on mammalian lifespan was quite encouraging; resveratrol improved insulin sensitivity, transcriptional profiles, and longevity in obese mice consuming a high-fat diet [5]. However, resveratrol appears to primarily protect from negative consequences of a high-fat diet, rather than ameliorating the underlying aging process, as two separate trials of dietary resveratrol, conducted by independent groups using multiple doses, failed to find any effect of resveratrol on lifespan of mice fed a standard lab diet [6,7]. Resveratrol has often been associated with the “French Paradox”, the observation that red wine consumption may ameliorate the deleterious effects of a high fat diet. While it is tempting to make this link, red wine contains many potentially beneficial molecules, and the concentration of resveratrol alone is likely too low to account for its benefits [1]. Nevertheless, resveratrol continues to show significant promise as a potential therapeutic, suppressing many forms of cancer, and improving insulin sensitivity, endurance, motor coordination, vascular tone, bone strength, and resistance to ischemic injuries in mice [1,5, 8–10].

There have been a number of significant controversies concerning the ability of resveratrol to extend lifespan in lower organisms, and its mechanism of action in mammals [11]. In both yeast and *Drosophila*, failure to replicate the original observation of lifespan extension has been reported [12,13]. However, lifespan extension has been reproduced in both organisms by the same groups that made the initial reports, and has also been reported independently [14,15]. A recent study in *Drosophila* has found that the effect of resveratrol on lifespan is dependent on the nutrient composition of the diet, suggesting that subtle differences in experimental conditions may be contributing to the discrepancies between findings from different labs [15]. While there is general agreement that resveratrol extends lifespan in *C. elegans* [16– 18], Bass et al. reported that the effect was variable and unrelated to the presence of Sir2 [12]. In contrast, Viswanathan et al. concurred with the original report that lifespan extension by resveratrol was robust and entirely dependent on Sir2 [19]. Therefore, a number of unresolved issues concerning the effects of resveratrol and their dependence on Sir2 remain to be clarified in lower organisms.

In mammals, resveratrol influences multiple direct targets, including cyclooxygenases [8], cytochrome P450 enzymes [20,21] the estrogen [22] and aryl hydrocarbon receptors [23] and quinone reductase 2 [24] and can indirectly activate the AMP-activated protein kinase (AMPK) [5,25] and Nrf2/Keap1 signaling pathways [26]. In addition, the biochemical evidence for direct activation of SIRT1 by resveratrol has been challenged by several groups, since the effect is dependent on the use of fluorescent substrates [13,27]. On the other hand, it is quite clear that many of the effects of resveratrol in cultured cells are dependent on the presence of SIRT1 [28–47] and the limited evidence that is available supports the same conclusion *in vivo* [48]. Therefore, key questions that remain to be resolved in mammals are whether SIRT1 activation by resveratrol occurs through a direct or indirect mechanism, and what the relative importance of this pathway is compared to other effects of the molecule that might contribute to its health benefits.

Notably, AMPK was recently shown to be required for many of the benefits of resveratrol in mice [49] and was previously shown to be required for lifespan extension by resveratrol in worms [18]. Mouse models lacking the catalytic subunits of AMPK fail to show increased insulin sensitivity, improved glucose tolerance, or enhanced mitochondrial biogenesis when treated with resveratrol, in contrast to wild-type animals [49]. Activation of AMPK alone appears to be sufficient to extend *C. elegans* lifespan [50] and is a possible explanation for the increased lifespan of mice lacking S6K1 [51]. Although SIRT1 can activate AMPK via deacetylation and activation of the upstream kinase LKB1 [52] activation of AMPK by resveratrol can occur independently from SIRT1 [53]. Thus, it remains unclear whether SIRT1 mediates the AMPK-dependent effects reported by Um et al. Unfortunately, the developmental and metabolic abnormalities in SIRT1 null mice, which are small and infrequently survive postnatally, have made it difficult to perform parallel studies to determine whether metabolic effects of resveratrol are similarly dependent on SIRT1 itself [54].

**Figure 1:**
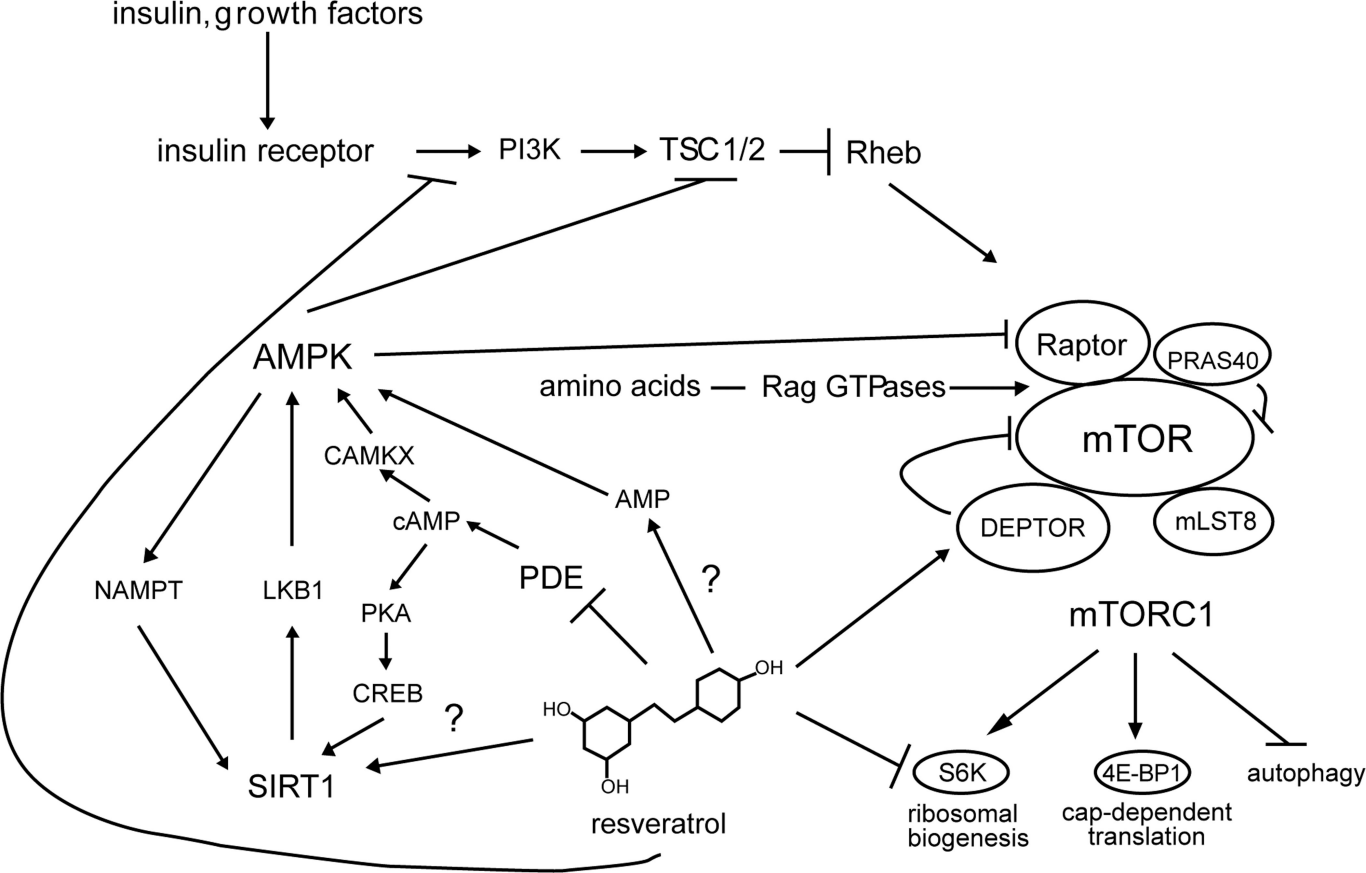
Resveratrol In addition to possible direct effects of resveratrol on SIRT1 activity, resveratrol also activates AMPK. It has been speculated that this might occur via inhibition of electron transport, with a consequent rise in ATP [5]. However, a recent report describes a mechanism by which resveratrol can cause AMPK activation via inhibition of cAMP-specific phosphodiesterases [59]. Resveratrol modulates protein translation via direction inhibition of S6K1 [159] and indirectly by promoting the association of mTORC1 with DEPTOR [160] and can acutely inhibit insulin signaling at the level of IRS1/2 and/or PI3K [161,162].

Interestingly, AMPK can also act upstream of SIRT1 by increasing production of its cosubstrate, nicotinamide adenine dinucleotide (NAD) [55] and Canto et al. have proposed that this is the major mechanism contributing to SIRT1 activation following resveratrol treatment *in vivo* [56]. One possible mechanism for SIRT1-independent AMPK activation by resveratrol is direct inhibition of mitochondrial oxidative phosphorylation, causing a drop in intracellular ATP levels, and consequently, a rise in AMP [57,58]. However, it remains to be seen if the concentrations of resveratrol achieved *in vivo* are sufficient to elicit this effect. During the preparation of this manuscript, Park et al. reported an alternative mechanism by which resveratrol can activate AMPK. Their study identified resveratrol as an inhibitor of cAMP-specific phosphodiesterases (PDEs), and described a multi-step mechanism by which increased cAMP leads to activation of AMPK via phosphorylation by CAMKK [59]. In addition, they showed that a specific inhibitor of PDE4, rolipram, is sufficient to reproduce many of the salient effects of resveratrol, including increases in glucose tolerance, endurance, and energy expenditure. Intriguingly, increased cAMP was also recently reported to lead to enhanced activity of SIRT1 through a separate, PKA-dependent mechanism [60]. Therefore, the ability of resveratrol to increase intracellular cAMP signaling provides a plausible upstream mechanism for the induction AMPK- and SIRT1-depdendent benefits (Figure 1).

A significant piece of evidence arguing in favor of sirtuins mediating key protective effects of resveratrol in mice is a series of studies involving SRT1720, a novel synthetic activator of SIRT1 [61]. Intriguingly, SRT1720 extends the survival and healthspan of mice fed a high-fat diet, just as resveratrol does [62]. In addition, this molecule has been shown to improve insulin sensitivity and endurance, and induces a transcriptional profile that is very similar to the effect of resveratrol [62–64]. Unlike resveratrol, SRT1720 does not have any acute effect on AMPK activity. However, long-term (> 20 weeks) treatment *in vivo* does result in modest AMPK activation [63]. Whether this is a direct result of increased SIRT1 activity, or instead indicates additional mechanisms of action for SRT1720 remains to be seen. Notably, SIRT1 activation by SRT1720, like activation by resveratrol, is substrate-dependent [65] and it has been suggested that inhibition of the acetyltransferase p300, rather than activation of SIRT1, may underlie some of SRT1720’s effects [66]. However, SRT1720 has been shown to increase SIRT1 activity *in vitro* using a substrate that contains only natural amino acids, supporting the model that its effects on SIRT1 activity *in vivo* are direct [67]. Therefore, structurally unrelated compounds that activate SIRT1 *in vitro* have similar protective effects *in vivo*, but further study will be required to reach a consensus concerning their *in vivo* mechanism(s) of action.

Resveratrol is currently being investigated in human clinical trials as an anti-cancer and anti-diabetic therapy, and is also marketed as a nutritional supplement with a variety of claims related mainly to weight loss and increased energy [68]. Although only a small fraction of the ongoing studies have been published in peer-reviewed journals to date, there is evidence that resveratrol can increase cerebral blood flow, improve insulin sensitivity, decrease inflammation, and suppress cardiovascular risk factors in humans [69–73]. A direct extrapolation from mouse studies, based on body weight, would suggest that humans would require a large quantity of resveratrol to obtain similar benefits, well above the ~1g/day at which significant gastrointestinal side effects have been reported [74]. As has been pointed out, however, scaling by body weight is not an accurate method for determining dosing across species [75]. The wisdom of this assertion was recently highlighted by the finding that 150 mg/day resveratrol in humans (~2 mg/kg/day) achieved equivalent or higher serum levels of resveratrol than 400 mg/kg/day in mice [73]. Subjects in the study exhibited reduced metabolic rates, improvements in glucose and lipid metabolism, and reduced inflammation, leading the authors to conclude that the effects of resveratrol resembled those of calorie restriction. While a number of safety trials in healthy humans have not raised any major concerns, it is noteworthy that a high-dose (5 g/day) trial of a resveratrol-based drug, SRT501, in multiple myeloma patients was halted due to kidney complications [76]. It was suggested that this effect was secondary to dehydration due to diarrhea, which generally does not occur at doses below 1 g/day. Therefore, the existing data in humans suggest that resveratrol supplementation may lead to improvements in health, and is likely to be safe. However, it will be of great importance to design future trials of resveratrol to detect adverse effects as well as potential benefits, and to continue to perform well-controlled studies to determine how much of the promise of this drug in mice will translate to human patients.

## Rapamycin

Rapamycin is an inhibitor of the mTOR (mechanistic Target Of Rapamycin) signaling pathway, which is found in most eukaryotes, including yeast, worms, flies, plants, mice, and humans. mTOR integrates inputs from nutrients and growth factors, including amino acids, glucose and insulin, to regulate many outputs involved in growth and proliferation [77]. Indeed, mTOR is so centrally positioned that it may be accurately described as a master regulator of cell metabolism.

mTOR is found in two distinct protein complexes: mTORC1, which regulates numerous cellular processes related to growth and differentiation, and mTORC2, which plays a regulatory role in the insulin signaling cascade, among other functions. Genetic attenuation of mTORC1 signaling is sufficient to promote longevity in diverse organisms, including *S. cerevisiae, C. elegans*, and *D. melanogaster* [78–80]. mTORC1 is the canonical target of rapamycin, whereas mTORC2 is not acutely sensitive to the drug. Rapamycin is FDA-approved as an immunosuppressant for transplant surgery, and is also being investigated for its anti-tumor properties. Interestingly, the immunological effects of rapamycin are proving more complex than initially supposed; the drug even enhances immunity under some conditions [81]. Studies conducted over the last few years have shown that rapamycin treatment can extend lifespan in model organisms, including yeast and flies [82–84] and that rapamycin treatment can even extend the lifespan of mice. In a study conducted by the National Institute on Aging Interventions Testing Program, rapamycin was found to extend the average and maximal lifespan of both male and female mice, even when treatment was initiated at 20 months of age [85]. A follow-up study demonstrated similar effects when rapamycin was begun at 9 months of age [7]. It has been suggested that rapamycin may extend rodent lifespan via an anti-tumor mechanism, however, the available data do not support a dramatic change in the range of cancers or other lethal or non-lethal illnesses found in the mice at the time of death. Instead, it is believed that rapamycin acts via an anti-aging mechanism, as suggested by the extension of both median and maximal lifespan and the delayed appearance of age-associated pathologies. These results strongly implicate the mTORC1 pathway in the regulation of mammalian longevity, and suggest that pharmacologic inhibition of mTOR signaling explains, or at least contributes to, lifespan extension by rapamycin.

The downstream mechanism by which mTORC1 inhibition extends lifespan is not yet clear. Rapamycin is an inhibitor of mTORC1-dependent translation, and one theory of aging suggests that decreased translation can extend lifespan by reducing the burden on the protein folding machinery, leading to improved protein quality. Indeed, genetic depletion of ribosomal proteins, as well as inhibition of translation initiation factors, can similarly extend lifespan in yeast and worms [86,87]. Moreover, deletion of the mTORC1 substrate S6 kinase 1 (S6K1), which plays a key role in the control of protein translation, is sufficient to confer increased lifespan in female mice [51]. However, from a quantitative standpoint, while rapamycin does decrease translation [88] the effect is mild *in vivo*, and the more salient effect may be a shift in the type of mRNA that is translated [89]. Moreover, the S6K-null mouse has no overt change in total translation in skeletal muscle, providing additional evidence that decreased translation *per se* may not be how mTOR inhibition promotes longevity [90]. In yeast, interfering with components of the 60S, but not the 40S subunit of the ribosome extends lifespan, and this correlates with increased translation of a specific mRNA encoding the transcription factor Gcn4, which is, itself, sufficient to extend life [87]. Whether a similar process might be occurring in mammals is not yet known. Clearly, much remains to be understood about the consequences of long-term mTORC1 inhibition *in vivo*.

Another possibility is that inhibition of mTORC1 may promote lifespan by inducing autophagy, the process responsible for the normal degradation and renewal of cellular components and organelles. A number of interventions that induce autophagy have been shown to extend the lifespans of model organisms, and the role of mTORC1 in suppressing autophagy is well-established [91,92]. Indeed, rapamycin treatment induces autophagy *in vivo*, reducing levels of amyloidbeta and rescuing cognitive defects in a mouse model of Alzheimer’s disease [93]. Therefore, induction of autophagy appears to account for at least some of the beneficial effects of rapamcyin, and other mTORC1-specific kinase inhibitors may have similar protective effects [92]. In addition, screens of FDA-approved compounds for regulators of autophagy may be of significant use in identifying molecules that could inhibit mTORC1 signaling in humans. In fact, one such screen has already identified four mTORC1-specific inhibitors - perhexiline, niclosamide, rottlerin and amiodarone (all likely acting through indirect mechanisms) - that activate autophagy [94]. In support of the potential importance of autophagy in preventing age-related decline, the restoration of autophagy to youthful levels in aged liver has been shown to reverse functional deficits [95]. Induction of autophagy may therefore have benefits in the treatment of age-related diseases that go well beyond it’s reported effects in Alzheimer’s disease models.

Interestingly, resveratrol also activates autophagy in at least some cell types [96–98] and in a cellular model of Parkinson’s disease, resveratrol provides a protective effect via increased autophagy through and AMPK and SIRT1 dependent mechanism [99]. However, resveratrol can also inhibit autophagy in a number of settings both *in vitro* and *in vivo* [100,101]. Although the mechanism accounting for these discrepancies is not yet clear, it may be that resveratrol stimulates opposing pathways, since the activation of autophagy is thought to proceed via SIRT1 [99], while inhibition appears to be due to suppression of signaling through S6K1 [100]. Therefore, the balance of resveratrol’s effects on these two enzymes may determine whether it activates or inhibits autophagy in a given cell type.

Caloric restriction (CR), a reduction in energy intake in the absence of malnutrition, extends mammalian lifespan [102,103] and decreases mTORC1 signaling in multiple tissues including liver, mammary tissues, and epithelia [104–106]. In yeast, replicative lifespan extension induced by rapamycin or interference with the TOR pathway is not additive with CR (glucose restriction), suggesting a common mechanism [78]. One of the hallmarks of CR in mammals, including humans, is a reduction in blood glucose and insulin levels, and increased insulin sensitivity [107]. Many long-lived mouse models, including the S6K null mouse, share these phenotypes, which have been postulated to contribute to lifespan extension [108,109] although mice lacking the insulin receptor substrate proteins IRS1 or IRS2 prove this is not a universal rule [110,111]. Conversely, mice fed a high-fat diet become glucose intolerant, insulin-resistant, and have a decreased lifespan [5]. These correlations make it somewhat surprising that rodents treated with rapamycin, despite extended lifespan, exhibit impaired glucose tolerance and insulin resistance, caused in part by increased hepatic gluconeogenesis [112,113]. In fact, humans treated clinically with rapamycin as an immunosuppressant have decreased insulin sensitivity and an increased incidence of type 2 diabetes [114,115]. These findings suggest that either rapamycin and CR work through distinct mechanisms, or that lifespan extension by CR is not directly related to improvements in insulin sensitivity.

**Figure 2:**
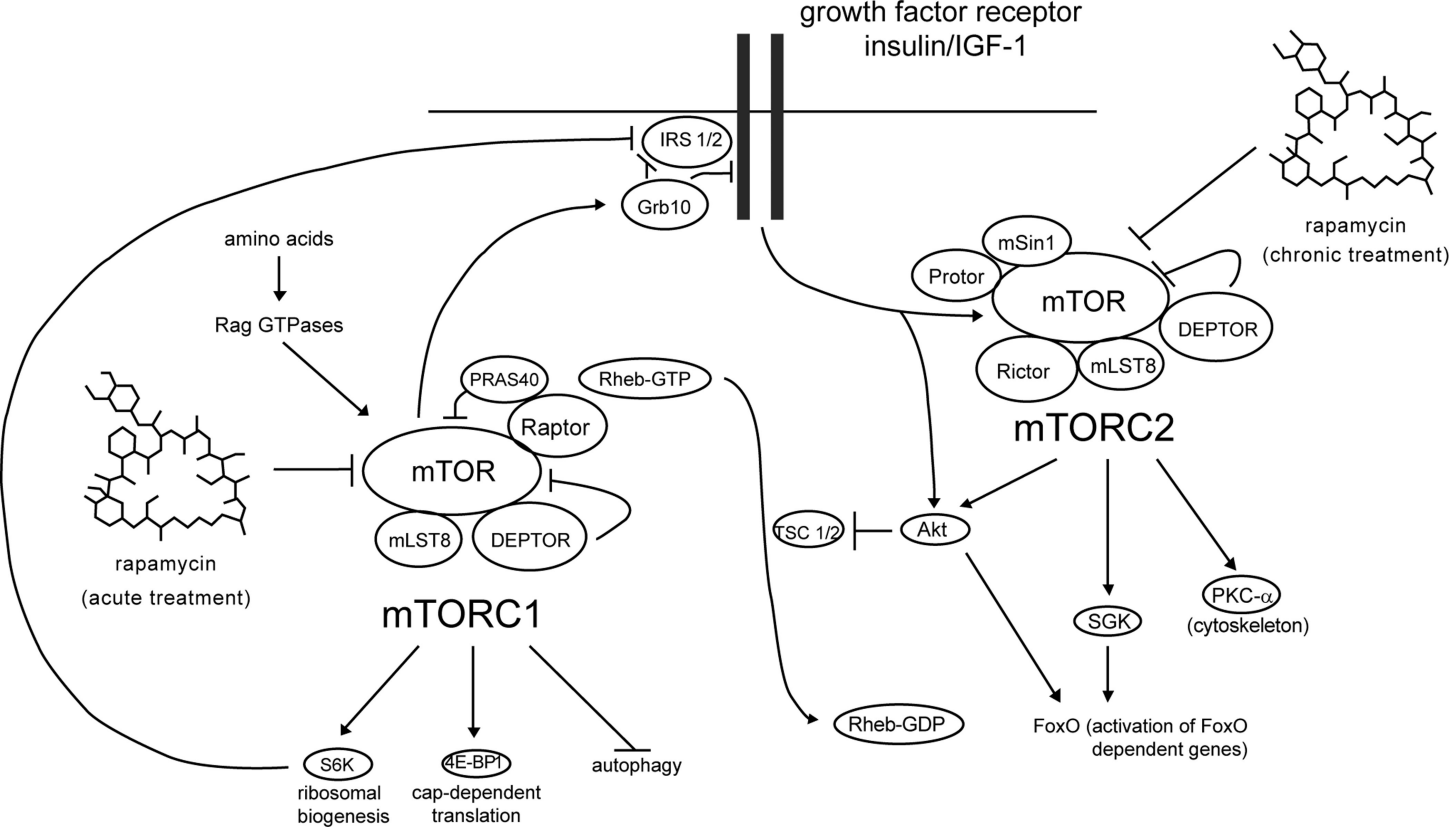
Rapamycin Acute treatment with rapamycin directly inhibits mTORC1, blocking the phosphorylation of S6K1 and 4E-BP1. mTORC1 signaling causes feedback at the level of the insulin receptor, mediated in part by S6K through phosphorylation of IRS1/2 [163] and in part by Grb10 [164]and relieving this inhibition enhances insulin signaling to mTORC2. Chronic treatment with rapamycin inhibits mTORC2 indirectly, most likely by interfering with its the assembly [116] resulting in the decreased signaling to the mTORC2 substrates Akt, PKCα and SGK.

While acute treatment with rapamycin specifically inhibits mTORC1 signaling, we have found that chronic rapamycin exposure over the course of 24–48 hours can inhibit mTORC2 in some cultured cell lines (Figure 2) possibly by preventing the assembly of new complexes [116]. We have recently demonstrated that this also occurs *in vivo* in each of the tissues that we have tested (liver, skeletal muscle, and white adipose), at the same doses that extend lifespan, within two weeks of treatment [117]. We have found that mTORC2 disruption is a major cause of insulin resistance induced by chronic rapamycin treatment *in vivo*. Therefore, at least some detrimental effects of rapamycin might be separable from mTORC1-dependent lifespan extension. This was exemplified by our recent finding that female mice heterozygous for both mTOR and mLST8 have reduced activity of mTORC1, but not mTORC2, and have increased longevity with normal glucose tolerance [117]. This strongly suggests that specific inhibitors of mTORC1 signaling may provide some of the benefits of rapamycin with respect to health and longevity, while avoiding sideeffects caused by inhibition of mTORC2. Interestingly, a number of the FDA-approved compounds identified by Balgi et al. [94] as inducers of autophagy that act via inhibition of mTORC1 do not appear to inhibit signaling to mTORC2, and thus may be interesting candidates to pursue in this regard.

The effect of mTOR and mLST8 heterozygosity on longevity was not observed in males, even though rapamycin extends life in both genders. Interestingly, rapamycin treatment has a stronger effect on lifespan in females than males [7]. Moreover, lifespan extension resulting from deletion of the mTORC1 substrate S6K1 is also specific to females [51] raising the possibility that lifespan extension by rapamycin in males is due to a separate mechanism. Soukas et al. recently showed that mTORC2 disruption confers lifespan extension in *C. elegans* fed a nutrient-rich diet, although it has the opposite effect under standard conditions [118]. In addition, inhibition of mTORC2 may contribute to the tumor-suppressive effects of rapamycin in humans [119]. Therefore, despite its negative effects on metabolism, mTORC2 disruption might also contribute to the overall improvement in longevity in rapamycin-treated mice. Treatment of humans with rapamycin has various other side effects, including altered testosterone and luteinizing hormone levels and, in males, decreased sperm production [120]. Gaining a more mechanistic understanding of the beneficial and detrimental effects of rapamycin should be a high priority for the aging research community, given its success at extending life in mice and its possible unsuitability for sustained use in healthy humans.

## Metformin

Metformin is an oral anti-diabetic drug that has been FDA approved since 1995, and is the consensus choice of the American Diabetes Association and the European Association for the Study of Diabetes as the initial pharmacologic therapy for hyperglycemia in type 2 diabetes [121]. Treatment with metformin lowers blood glucose levels, inhibits lipolysis and decreases circulating free fatty acids, while producing few undesired side effects [122]. Although metformin may have a protective effect on β-cells, it does not directly affect insulin secretion [123]. Instead, metformin treatment results in increased insulin sensitivity in the liver and muscle, resulting in decreased hepatic gluconeogenesis and increased peripheral utilization of glucose [122]. Metformin is also effective in preventing high-risk individuals from developing type-2- diabetes [124]. Long-term follow-up of patients from studies including the UK Prospective Diabetes Study has shown that the treatment of diabetic patients with metformin decreased mortality from all causes, including diabetes-related mortality, cancer, and myocardial infarction [125,126].

**Figure 3:**
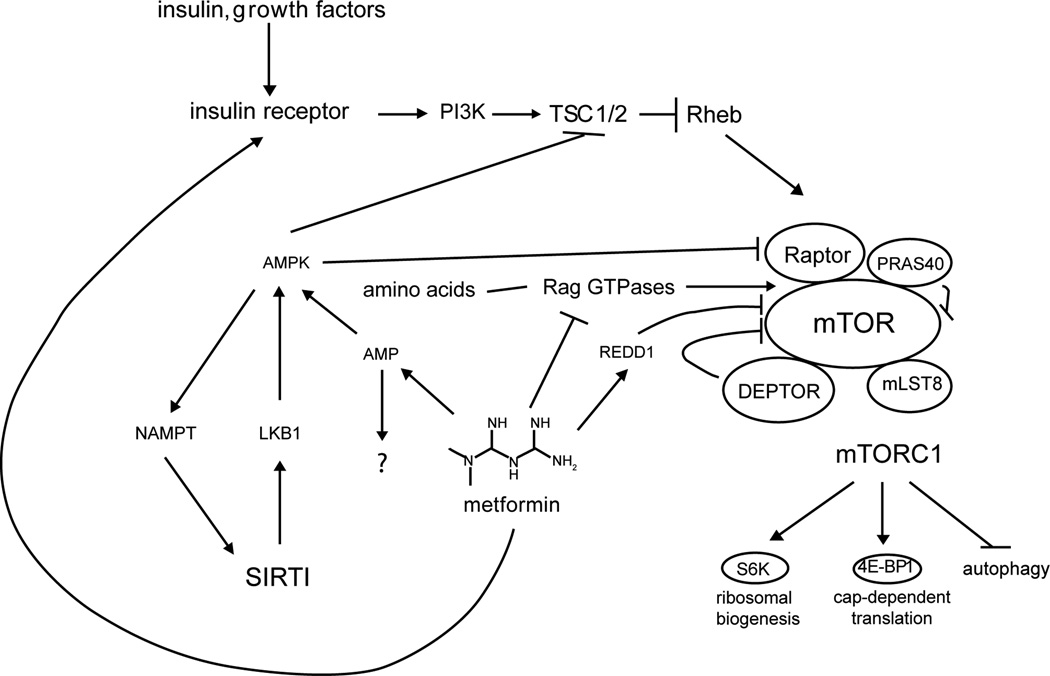
Metformin Metformin inhibits complex I of the electron transport chain, leading to a rise in intracellular AMP and activation of AMPK [131,132]. AMPK activation can inhibit mTORC1 activity via phosphorylation of TSC2 [136] and Raptor [137] but metformin also independently inhibits the Rag family of GTPases that regulate mTORC1 localization [139]. Notably, the suppression of hepatic glucose output by metformin was recently shown to be dependent on reduced energy charge, but independent of AMPK [133]. Metformin further enhances insulin sensitivity directly at the level of the insulin receptor [128] and likely has indirect effects through mTORC1 by regulating the stability of IRS1/2 and Grb10.

The molecular mechanism(s) underlying the actions of metformin have been difficult to pin down [127]. A number of antidiabetic effects have been reported, including activation of the insulin receptor [128] and stimulation of the incretin axis [129] but most attention has focused on metformin’s role as an activator of AMPK [130]. As a central regulator of energy balance within the cell, activated AMPK induces a complex series of changes that result in an overall decrease in anabolic processes and enhancement of catabolic processes to restore ATP levels. While it is undisputed that metformin activates AMPK *in vivo*, metformin does not activate AMPK *in vitro*, demonstrating that the mechanism of action is indirect. Accumulating evidence suggests that AMPK activation during metformin treatment may, in fact, be secondary to direct energetic stress, caused by inhibition of complex I of the mitochondrial respiratory chain and a subsequent fall in the ADP:ATP ratio with an accompanying rise in AMP [131,132]. Moreover, inhibition of hepatic gluconeogenesis, which is perhaps the most clinically relevant effect of metformin, appears to be mediated by energetic stress through an AMPK-independent mechanism [133]. Therefore, AMPK activation may well be a bystander in many of metformin’s effects, rather than a central player in mediating improvements in metabolism. Interestingly, inhibition of complex I is not observed when metformin is supplied directly to isolated mitochondria, but the effect is restored when metformin is supplied in lysosomal form, suggesting that a membrane-mediated event is required [134]. Elucidating this mechanism, and the downstream events that lead to suppression of hepatic glucose output, will be major challenges for the field in the coming years. (Figure 3)

Importantly, treatment with metformin inhibits the mammalian target of rapamycin (mTOR) signaling pathway, resulting in decreased phosphorylation of the mTOR complex I (mTORC1) substrates S6K1 and 4E-BP1 and decreased translation [135]. These effects of metformin on mTORC1 activity were presumed to result entirely from activation of AMPK, since AMPK-dependent phosphorylation activates TSC1/2, a repressor of mTORC1 activity [136], and inhibits raptor, a component of mTORC1 [137]. However, it was recently demonstrated that metformin can regulate mTORC1 independently from AMPK via induction of the mTORC1 inhibitor REDD1 [138], and separately, via regulation of the Rag GTPases [139]. The Rag GTPases regulate the localization of mTORC1 in response to amino acids, and are required for mTORC1 activity [140,141]. There is also evidence that metformin can regulate autophagy, either through inhibition of mTORC1 signaling or via an AMPK-dependent pathway. Metformin has been shown to activate autophagy in cardiac cells [142] as well as in melanoma [143]. Together, these results suggest that the effects of rapamycin and metformin may be partially overlapping.

It has also been proposed that metformin can suppress gluconeogenesis through altering the balance of acetylation on key transcriptional regulators, which is mediated largely by SIRT1 and GCN5 [144]. Here again, an AMPK-dependent mechanism is likely to contribute, since AMPK activation enhances expression of nicotinamide phosphoribosyltransferase (Nampt), and thereby increases the availability of NAD, a cosubstrate for SIRT1 [55]. However, increases in GCN5 mRNA, and GCN5 and SIRT1 protein levels were all found to be independent of AMPK in this study [144].

Based on its ability to reduce circulating glucose, insulin, and IGF-1, and to disrupt signaling from the latter hormones to mTORC1, metformin has been suggested as a potential CR mimetic and anti-aging compound. Transcriptional profiling supports a significant overlap between the effects of metformin and CR [145] and metformin extends both the lifespan and healthspan of the nematode *C. elegans* [146]. In *C. elegans*, the effects of metformin on lifespan are independent of the insulin signaling pathway, but are dependent on AMPK and LKB1, as well as the oxidative stress transcription factor Skn-1/Nrf2 [146]. Skn-1 [147] and AMPK [148,149] are also required for certain CR regimens to extend the lifespan of *C. elegans*, suggesting that CR and metformin may extend lifespan by similar mechanisms in this organism. Interestingly, Skn-1/Nrf2 has also been implicated in the lifespan extension induced by inhibition of translation, raising the possibility that it might also be active during inhibition of the TOR signaling pathway [150].

With respect to mammals, metformin was shown to extend the lifespan of short-lived tumor-prone HER2/neu mice [151]. In addition, a study that looked at 50 control vs. 50 metformin-treated (100 mg/kg/day) female SHR outbred mice found a 91.9% increase in median lifespan, a 37.8% increase in mean lifespan, and a 10.3% extension in maximum lifespan [152]. Unfortunately, the interpretation of this study with respect to aging is compromised by the tumor susceptibility and short lifespan of SHR mice. In contrast, a recently completed study on the metformin treatment of Fisher-344 rats (300 mg/kg/day) failed to show increased lifespan [153]. The reason for the disparity between this study and the study by Anisimov et al. [152] is unclear, but could be related to the differences in species (mice vs. rats), the higher dose of metformin used by Smith et al. [153], or the tumor susceptibility of the strains used by Anisimov et al. [152]. In a mouse model of Huntington’s disease, it has been reported that while treatment with a high dose of metformin (750 mg/kg/day) had no effect on survival, a lower dose (300 mg/kg/day) significantly extended lifespan [154]. A similar dose response was seen in a recent *C. elegans* study, in which a high dose of metformin (100mM) failed to extend lifespan while a lower dose (50mM) succeeded [146]. The study by Smith et al. [153] also had technical issues that make it difficult to interpret the results. With respect to the biological activity of the metformin, the treated rats in the Smith et al. [153] study failed to displayed significantly altered glucose or insulin levels, which are well-established consequences of metformin treatment. In addition, Smith et al. [153] included a CR group as a control; this group had a statistically insignificant increase in lifespan of only 8.7% on a 30% CR diet. Further, the CR rats had lower insulin levels than the ad libitum controls at only one out of four time points. The authors of the study propose that that the diet fed to these rats, NTP-2000, may explain some of these discrepancies, but the failure of the CR diet to extend lifespan means that the effects of metformin on longevity are not conclusive [153].

Given the potential of metformin treatment to recapitulate effects of resveratrol, rapamycin, and CR, it will be extremely interesting to see whether it can influence longevity in a long-lived strain of mice, such as C57BL/6. Unlike resveratrol and rapamycin, the use of which in humans has been generally very limited, metformin is regarded as a well-tolerated and affordable compound that is currently being administered to millions of patients. Therefore, there is a major opportunity to study the incidence of age-related diseases in patients who are already taking metformin, which could provide valuable evidence as to its efficacy for preventing age-related diseases prior to the initiation of lengthy and expensive clinical trials.

## Conclusion

Modulation of the rate of aging in mammals is achievable, as is routinely demonstrated by studies of caloric restriction and genetic manipulation in rodent models. Given the potential benefit in terms of amelioration of age-related diseases, the search for pharmacologic means to mimic these effects should be a high priority in biomedical research. As such, determining the mechanism by which anti-aging compounds truly impact lifespan is of significant importance for pharmaceutical development. While rapamycin, metformin, and resveratrol all exert beneficial effects on health that could conceivably influence human longevity, the molecular mechanisms by which they function remain unclear.

The compounds discussed in this review highlight the difficulties in studying a complex phenotype such as aging. While some progress has been made, it will take a concerted effort by many groups to assign definitive mechanisms and refine approaches to translate the benefits seen in rodents to the human population. Although this review has been focused on three specific compounds, there are many other promising molecules whose mechanisms of action require further study, e.g. spermidine [155] aspirin [156] l-deprenyl [157] oxaloacetate [158] and a variety of antioxidant and anti-inflammatory compounds. In particular, it will be important to design future studies that test combinations of different approaches in order to determine whether a common downstream effect of various treatments explains most changes in longevity, or if multiple factors contribute to lifespan independently, and might have additive effects. Finally, as is highlighted by the effects of resveratrol and metformin on murine lifespan, the influence of factors such as diet and strain background must be carefully considered when interpreting studies of longevity. While any molecule that measurably improves health in aging mice potentially provides an exciting lead for the development of human therapeutics, an intervention that truly slows the underlying aging process should extend the maximum survival time of lean, healthy animals on a long-lived strain background. Given the potential benefit to human health, identifying and understanding genetic and pharmacologic interventions that pass this test should be a major focus for biomedical research.
